# Prevalence, vulnerability and epidemiological characteristics of snakebite in agricultural settings in rural Sri Lanka: A population-based study from South Asia

**DOI:** 10.1371/journal.pone.0243991

**Published:** 2020-12-28

**Authors:** Subashini Jayawardana, Carukshi Arambepola, Thashi Chang, Ariaranee Gnanathasan

**Affiliations:** 1 Faculty of Medicine, Department of Allied Health Sciences, University of Colombo, Colombo, Sri Lanka; 2 Faculty of Medicine, Department of Community Medicine, University of Colombo, Colombo, Sri Lanka; 3 Faculty of Medicine, Department of Clinical Medicine, University of Colombo, Colombo, Sri Lanka; Universidad de Costa Rica, COSTA RICA

## Abstract

**Background:**

The burden of snakebite remains poorly characterised because of the paucity of population-based data. Further, factors determining the vulnerability of individuals within rural communities to snakebite have been rarely investigated. We undertook a population-based study to determine the prevalence, vulnerability and epidemiological characteristics of snakebite in rural Sri Lanka.

**Methods and findings:**

A population-based cross-sectional study was conducted among 8707 current residents in the district of Ampara, representing typical rural Sri Lanka. The sample was recruited using multi-stage cluster sampling with probability proportionate-to-size. Snakebite victims were identified using the WHO criteria. Data were collected using a pre-tested interviewer-administered questionnaire.

Each household had on average 3.8 persons; mean age 28.3 years (SD = 18.2); 51.3% males. The one-year point prevalence of snakebites was 17.6 per 1000 residents (95% CI: 15–20.6) and 6.12 per 100 households (95% CI: 5.25–7.13), while the lifetime prevalence was 9.4 per 100 residents (95% CI: 8.8–10.0) and 30.5 per 100 households (95% CI: 28.6–32.2) with a case fatality ratio of 0.033. Venomous snakebites accounted for 28.1%; snakes were unidentified among 30.1%. Compared to the non-snakebite victims, being single, males, of Sinhala ethnicity, aged >19 years, low education and socioeconomic status, engaging in farming or unskilled outdoor occupations denoted vulnerability to snakebites. Outdoor bites (77.8%) were more common among males; during daytime; mostly while walking; within the rural terrains and home gardens; on lower limbs; mostly by hump-nosed and Russell viper. Indoor bites were more common among females; during night-time; while sleeping and barefooted; on lower limbs; mostly by hump-nosed vipers, kraits and non-venomous snakes.

**Conclusions:**

The burden of snakebite is considerably high among rural populations. The concept of vulnerability can be useful in healthcare decision-making and resource allocation.

## Introduction

Snakebite is a neglected tropical disease with an estimated 1.2–5.5 million snakebites per year resulting in 0.42 million envenomings and 0.02 million deaths worldwide [1]. Snakebite is a common medical emergency especially among rural residents of tropical and subtropical countries [2], particularly in South Asia, Southeast Asia and sub-Saharan Africa [1,3–6].

Sri Lanka, a tropical South Asian island nation, has 105 terrestrial snake species, of which six have been identified as medically important because of their venomosity [7]. Sri Lanka is one of the highest envenoming reporting countries in the region. A recent island-wide survey reported an estimated incidence of over 80,000 bites, 30,000 envenomings and 400 deaths per year, predominantly among rural residents engaged in agriculture related occupations [8].

In rural areas of Sri Lanka, snake envenoming is higher in the dry agro-climatic zone than in other zones, owing to the abundance of natural habitats of flora and fauna that provide shelter to the venomous snakes [9]. The biting habits and circumstances of snakebites vary according to the species of snake and its geographical distribution [10,11]. Cobra *(Naja naja)* is usually encountered during daytime in and around rural households [12]; Russell viper (*Vipera russelli*) in paddy fields at any time of the day; common krait (*Bungarus caeruleus*) indoors at night; and saw-scaled viper *(Echis carinatus)* in areas where vegetation is overgrown [11–16]. In contrast, Ceylon krait (*Bungarus ceylonicus*) which is indigenous to Sri Lanka is found mostly in the wet zone while hump-nosed viper (*hypnale hypnale*) is prevalent in all agro-climatic zones in the tropics, but particularly abundant in tea, rubber and coconut plantations [16,17]. Venomous snakes are usually encountered in sub-standard housing conditions or as an occupational hazard during agricultural work in paddy fields, chena cultivation and forestry. Owing to this wide variation in snake habitats and biting habits, distinct differences may exist within rural areas in relation to the individuals more at risk of snakebites compared to those who are not. Furthermore, snakes responsible for indoor bites are likely to differ from those responsible for outdoor bites. Therefore, even within a region at high risk for snakebites, there is likely to be a variation in the vulnerability of different population sectors for snakebite, which is determined by the biting habits and circumstances of the snakebite.

Despite the devastating fatal and long-term outcomes [18–20], snakebite has remained largely a neglected tropical condition, with its vulnerability among those living in specific geographical regions not being adequately explored. Unless the extent of this public health issue is well-recognised based on evidence, prioritising resource allocation and services for effective prevention and management would be challenging.

Previous studies from Sri Lanka report crude overall community incidence of snakebite based on projections calculated for populations using mathematical models [8], while estimates are also made based on samples predominantly from hospital attendees [16], which are likely to have underestimated the true prevalence of snakebite because a substantial proportion of snakebite victims seek indigenous remedies rather than present to hospital. Although these surveys have provided vital information on the overall incidence as well as epidemiological characteristics of the victims in Sri Lanka, the risk imparted by each characteristic for ever becoming a victim (victim versus non-victim) or the circumstantial variations related to bites (indoor versus outdoor) within snakebite-dense rural areas have not been well captured. Accurate epidemiological characterisation of snakebites as well as knowledge on specific vulnerabilities of residents particularly within rural communities are required to draw the immediate attention of healthcare providers and policy-makers to develop effective prevention of snakebite specific to each region within the country as well as to plan effective national and global campaigns against snakebites. Studies providing such representative data are sparse. Therefore, we conducted a population-based study to estimate the prevalence, vulnerability and epidemiological characteristics of snakebite in an area typically representative of the rural settings in Sri Lanka.

## Material and methods

A population-based cross-sectional study was conducted among the residents of Ampara District, which had been among the three districts reporting the highest number of snakebites over the last decade in Sri Lanka. It covers a land area of approximately 4,415 km^2^ and belongs to the dry agro-climatic zone in the country, which is well known for its rural agricultural environment conducive for snake habitats of natural water sources and forests [21]. The main occupation of residents is paddy, sugarcane and chena cultivation, depicting their relatively low socio-economic status. Owing to these outdoor occupations and rural terrain, Ampara District typically represents the rural agricultural communities most vulnerable to snakebites in Sri Lanka.

The study population included residents who have been living in the district for more than one year. Semi-permanent residents in commercial/work sites and permanent residents in religious institutes or elderly homes, and infants were excluded from the study. The minimum sample required for the study was 8470 residents, in order to detect an expected snakebite prevalence of 0.04 (based on a pilot survey carried out in the same district that reported three snakebite victims in the preceding 12 months among 75 residents) [22], with 0.015 precision at 5% level of significance. This sample also accounted for a design effect of 10.8 calculated for an intra-class correlation of 0.2 and cluster size of 50, and a non-response rate of 15%.

Ampara District (648,057 population) is divided into 20 divisional secretariat (DS) divisions and 508 grama niladhari (GN) divisions for administrative purposes [23]. From this sampling frame, the GN divisions identified as ‘urban’ (divisions under the administration of a municipal or urban council) were excluded, and the rest was taken as the sampling frame for the study. Next, assuming a minimum of seven eligible members per two households, 2500 households were selected using a systematic random sampling method (50 households per GN division x 5 GN divisions per DS division x 10 DS divisions), from which the chief householder and all eligible members belonging to the nuclear family were recruited to the study.

Following informed written consent, all eligible members in the selected households were recruited for the study by two trained investigators during house-to-house visits. A pre-tested questionnaire in the local vernacular languages (Sinhala and Tamil) was administered to collect data on the demographic and socio-economic characteristics of all household members, snake bites at both household and individual levels, and the characteristics of snakebite victims of the preceding year in relation to time, place and person.

A ‘snakebite victim’ was defined as any person who had been bitten by a snake during the preceding 12 months, five years or ever in life. The definition was further refined according to the evidence provided by the participants, to categorise the bite as follows:

- ‘Certain’—Availability of documental evidence confirming the snakebite, such as hospital clinic or diagnosis card; these records are usually kept with the patient
- ‘Probable’—No documental evidence but the snake had been seen by the victim and/or bystander at the time of the bite
- ‘Possible’—No documental evidence or snake seen, but the victim, bystanders and/or medical staff (as written in the diagnosis card) suspects that it was a snakebite

This working definition is keeping with the WHO Guidelines for the Management of Snakebites SEARO [24]. The bites were further confirmed by showing pictures of the common snakes.

Ethics clearance for the study was obtained from the Ethics Review Committee of the Faculty of Medicine, University of Colombo, Sri Lanka.

### Data analysis

Point prevalence of snakebite was calculated in relation to being a snakebite victim during the following three time points: in the preceding 12 months, last five years and lifetime. This prevalence was assessed at individual level as a proportion per 100 rural residents; and at household level as a proportion per 100 rural households. For each, 95% confidence intervals (CI) were calculated. Further, incidence density of snakebite was calculated per 1000 persons per year using the following formula, as per used in previous studies [3]:

$$=\frac{No.\;of\;persons\;reporting\;of\;ever\;being\;a\;snakebite\;victim}{Total\;person‐years\;at\;risk\;of\;snakebites\;during\;lifetime}\;x\;1000$$

Total person-years at risk was assessed by multiplying the number sampled by their mean age in years.

Data were analysed using Statistical Package for Social Sciences (SPSS) Version 20. Quantitative data were described in mean and standard deviation (SD) and qualitative data in proportions. Factors associated with the vulnerability of rural residents to snakebite were assessed, by comparing the personal characteristics of the ever snakebite victims with those who had never experienced a snakebite using odds ratio (OR) and 95% CI in univariate analysis, and thereafter in logistic regression analysis. All the variables significant in the univariate analysis at 0.05 significance were included as independent variables in the model; and using backward LR regression method, the vulnerability for snakebite was assessed. Further, among the snakebite victims of the preceding 12 months, circumstances in relation to the time, place and person of the snakebite were assessed in victims with indoor and outdoor bites.

## Results

From the 2500 households selected as per the sampling protocol, 8,707 persons fulfilling the eligibility criteria were recruited for the study. 75% of their households had ≥ three members (3.8 residents on average per household). Mean and median age of the participants were 28.3 years (SD = 18.2) and 26 years, respectively. The sample was comparable with most of the demographic and socio-economic characteristics of the rural population in Sri Lanka.

### Prevalence of snakebite at household level (N = 2500)

Of the 2500 households sampled, those ever reporting a victim of snakebite was 763, giving a lifetime prevalence of 30.5 per 100 rural households (95% CI: 28.6–32.2). Victims of 25 of these households succumbed to their injury, giving a case fatality ratio of 0.033 at the household level (25/763). In further analysis, a total of 153 households claimed to have at least one victim during the preceding 12 months, giving a one-year point prevalence of 6.12 per 100 rural households (95% CI: 5.25–7.13); 13.8 during last five years; 5.4 during last 5–10 years; and 14.9 before 10 years. The household prevalence of ever snakebites ranged from 2 to 68 at GN division level, while the highest and lowest household prevalence was reported from the same DS divisions during last five years. On average, the number of ever snakebite victims per household was 1.2.

### Incidence and prevalence of snakebite at individual level (N = 8707)

The incidence density of snakebites was 3.3 cases per 1000 rural residents per year.

**Fig 1:**
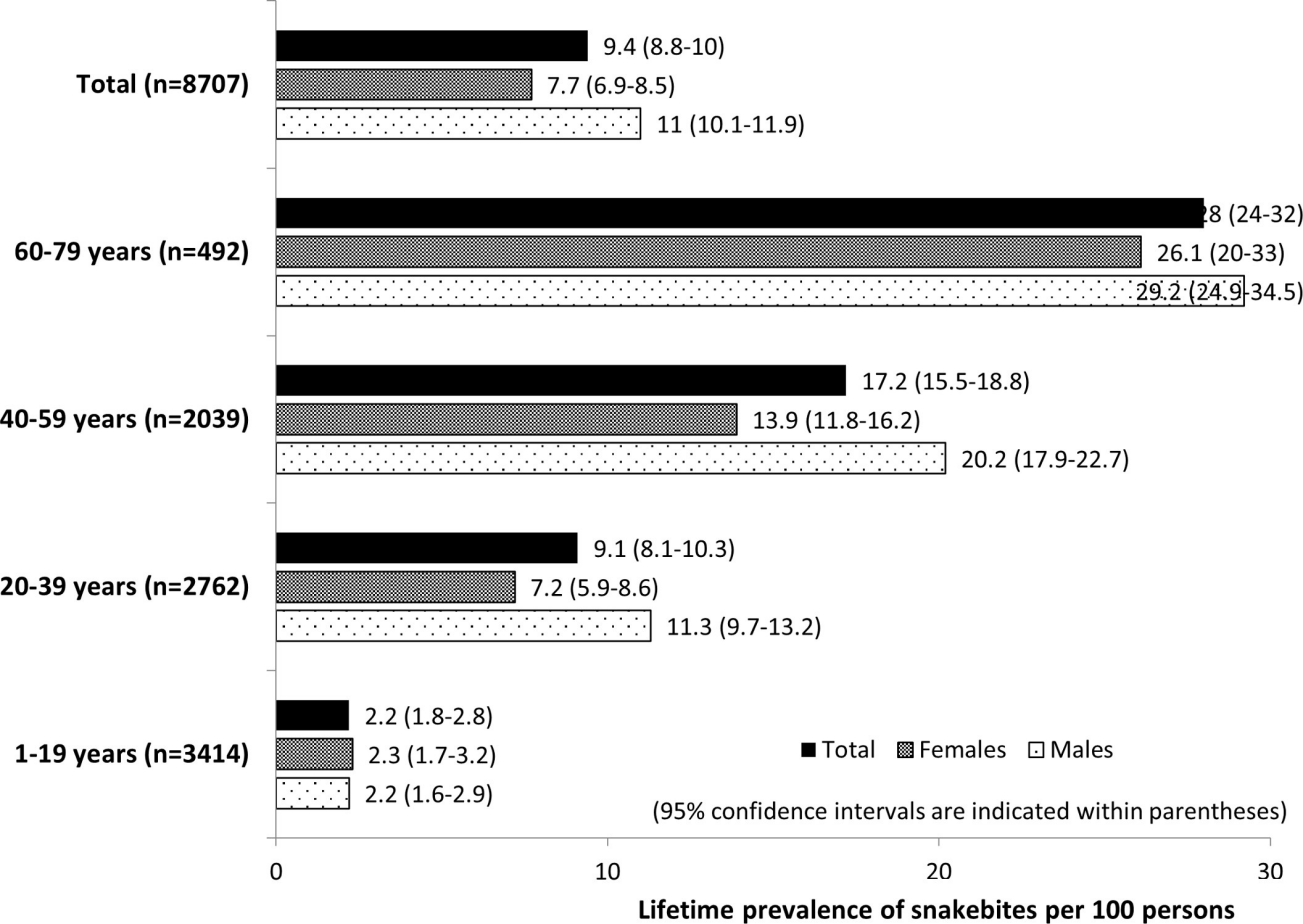
Age- and sex-specific lifetime prevalence of snakebite among rural residents in Sri Lanka.

The age- and sex-specific prevalence of snakebite during lifetime is shown (Fig 1). Among the 8707 rural residents sampled, 816 had ever been bitten by a snake at least once, giving a lifetime prevalence of 9.4 per 100 rural residents (95% CI: 8.8–10.0). Their first bite had been at the mean age of 35.3 years (SD = 15.6) occurring within a wide age range (1–77 years), while the sex distribution was 60% males: 40% females. Of the ever-bitten victims, 90.5% have been bitten by a snake only once, while it was twice in 6.8% and more than that in the rest, highlighting some degree of clustering of bites at individual level. Further, as expected, snakebite prevalence was higher with increasing age. When stratified by age groups, the prevalence was higher among males compared to the females in all except the 1-19-year age group that showed almost a similar prevalence. In both males and females, prevalence in the 60-79-year age group was nearly 10-fold higher compared to the least risk group of 1–19 age group.

**Fig 2:**
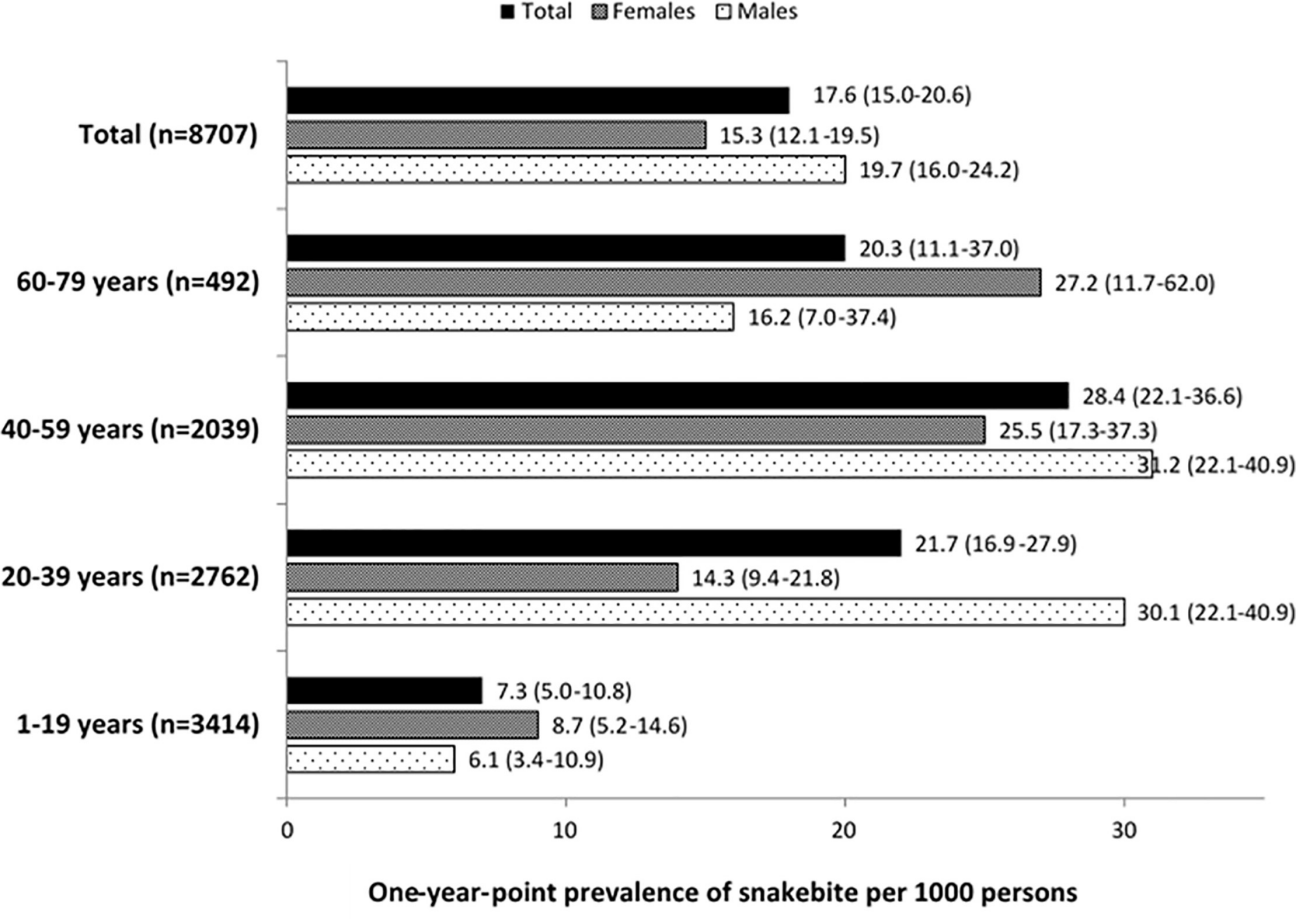
Age- and sex-specific point prevalence of snakebite in the preceding 12 months among rural residents in Sri Lanka.

The age- and sex-specific prevalence of snakebites in the preceding year is shown (Fig 2), showing a one-year point prevalence of 17.6 per 1000 rural residents (95% CI: 15–20.6). Mean age of the victims was 36.3 years (SD = 15.7). The highest prevalence among males was in the 40-59-year age group and in females, it was in 60-79-year age group. Compared to the least risk group of 1–19 age group, this prevalence was 3-fold higher amongst females and 5-fold higher amongst males.

### Vulnerability of the rural residents to snakebite

Factors associated with being vulnerable to snakebite among those who had ever experienced a snakebite compared to those who never had are presented (Table 1). Being a male, older age (over 19 years), being of Sinhalese ethnicity, currently not in marriage, engaging in farming or unskilled outdoor occupations, education below grade 6 and low income less than LKR 20,000 (USD 105) per month were the factors associated with being vulnerable to snakebite. After adjusting for confounders, all remained significant except the type of occupation (outdoor versus indoor).

**Table 1:** Vulnerability of the rural residents for snakebite in relation to their demographic and socio-economic characteristics (N = 8707).

Characteristic	No. ever snakebite victims (%) (n = 816)	No. non-victims (%) (n = 7891)	Crude OR (95% CI)	Adjusted OR (95% CI) ^3^
**Gender**				
Male	490 (11.0%)	3981 (89.0%)	1.48 (1.27–1.71)	1.66 (1.42–1.94)
Female	326 (7.7%)	3910 (92.3%)	1.00	1.00
**Age group**				
60 and above ^1^	138 (28.0%)	354 (72.0%)	7.23 (5.68–9.21)	5.44 (3.65–8.1)
40–59 years ^1^	350 (17.2%)	1689 (82.8%)		
20–39 years ^1^	252 (9.1%)	2510 (90.9%)		
1–19 years	76 (2.2%)	3338 (97.8%)	1.00	1.00
**Ethnicity**				
Sinhala	625 (10.7%)	5222 (89.3%)	1.68 (1.42–1.99)	1.69 (1.42–2.04)
Tamil ^1^	71 (6.1%)	1092 (93.9%)	1.00	1.00
Muslim/Moor ^1^	84 (6.4%)	1221 (93.6%)		
Other ^1^	36 (9.2%)	356 (90.8%)		
**Current marital status**				
Single ^1^	693 (14.9%)	3972 (85.1%)	5.88 (4.81–7.19)	2.15 (1.56–2.96)
Widowed ^1^	6 (54.5%)	5 (45.5%)		
Married	117 (2.9%)	3914 (97.1%)	1.00	1.00
**Highest level of education**				
No formal education ^1^	55 (5.7%)	917 (94.3%)	1.57 (1.36–1.81)	2.23 (1.91–2.61)
Grade 1–5 ^1^	339 (14.3%)	2026 (85.7%)		
Grade 6–10 ^2^	289 (6.8%)	3965 (93.2%)	1.00	1.00
Ordinary Level examination^2^	118 (11.8%)	885 (88.2%)		
Above Ordinary Level ^2^	15 (13.4%)	97 (86.6%)		
**Type of employment**				
Farming ^1^	516 (14.6%)	3020 (85.4%)	2.97 (2.55–3.46)	1.28 (1.05–1.55)
Unskilled work ^1^	26 (15.5%)	142 (84.5%)		
Skilled/Technical work ^2^	72 (14.7%)	418 (85.3%)	1.00	1.00
Student ^2^	67 (2.7%)	2437 (97.3%)		
Housewife ^2^	109 (12.5%)	765 (87.5%)		
None of the above ^2^	26 (2.3%)	1109 (97.7%)		
**Household income (Rs.)**				
10,000 or less ^1^	337 (13.7%)	2115 (86.3%)	1.65 (1.37–1.98)	1.46 (1.2–1.77)
11,000–20,000 ^1^	332 (8.3%)	3677 (91.7%)		
21,000–30,000 ^2^	124 (7.0%)	1644 (93.0%)	1.00	1.00
31,000–40,000 ^2^	13 (3.8%)	330 (96.2%)		
> 40,000 ^2^	10 (7.5%)	123 (92.5%)		

^1,2^ Categories combined for univariate and logistic regression analysis.

^3^ Model summary statistics: Overall percentage correctly identified 90.7%; Cox & Snell R Square: 0.071; Model significance: Chi-square value = 641.011; df = 7; p = 0.000.

### Circumstantial characteristics of the outdoor and indoor bites

The snake responsible for the bite in the preceding 12 months was identified with certainty in 51 cases, probable in 52 and possible in 4 victims; and not identified in 46. Of these, 43 were by venomous snakes (28.1%). There were 34 (22.2%) indoor bites and 119 (77.8%) outdoor bites. Their characteristics were further analysed in person, place and time (Table 2).

**Table 2:** Comparison of the outdoor and indoor snakebites bites by their person, place and time distribution in the preceding 12 months (n = 153).

Characteristics	Type of bite, No. (%)			Level of significance
	Indoor	Outdoor	Total	
**Sex**				
Males	12 (35.3%)	76 (63.9%)	88 (57.5%)	X^2^ = 8.8; df = 1; **p = 0.003**
Females	22 (64.7%)	43 (36.1%)	65 (42.5%)	
**Age category**				
1–19	9 (26.5%)	16 (13.4%)	25 (16.3%)	X^2^ = 1.5; df = 1; p = 0.2
20–39	13 (38.2%)	47 (39.5%)	60 (39.2%)	
40–59	10 (29.4%)	48 (40.3%)	58 (37.9%)	
60 and above	2 (5.9%)	8 (6.7%)	10 (6.5%)	
**Type of snake**				
Venomous				
*Russel viper*	1 (2.9%)	15 (12.6%)	16 (10.4%)	X^2^ = 0.45; df = 1; p = 0.5
*Krait*	2 (5.9%)	2 (1.7%)	4 (2.6%)	
*Hump-nosed viper*	5 (14.7%)	18 (15.1%)	23 (15.0%)	
Non-venomous	17 (50.0%)	47 (39.5%)	64 (41.8%)	
Not identified	9 (26.5%)	37 (31.1%)	46 (30.1%)	
**Site of the bite**				
Lower limb	25 (73.5%)	96 (80.7%)	121 (79.1%)	X^2^ = 0.8; df = 1; p = 0.4
Upper limb	8 (23.5%)	22 (18.5%)	30 (19.6%)	
Trunk	1 (2.9%)	1 (0.8%)	2 (1.3%)	
Footwear (n = 121) *				
Barefooted	24 (96.0%)	64 (66.7%)	88 (72.7%)	X^2^ = 13.1; df = 1; **p<0.01**
Wearing slippers	1 (4.0%)	32 (33.3%)	33 (27.3%)	
Clothing (n = 121) *				
Clothing up to ankles	1 (4.0%)	29 (30.2%)	30 (24.8%)	X^2^ = 7.9; df = 1; **p = 0.005**
Clothing up to knees	18 (72.0%)	27 (28.1%)	45 (37.2%)	
Clothing above knees	6 (24.0%)	40 (41.7%)	46 (38.0%)	
**Place of occurrence**				
Inside house	32 (94.1%)	-	33 (21.6%)	
In the home environment	1 (2.9%)	45 (37.8%)	46 (30.1%)	
In the paddy field/chena	-	27 (22.7%)	27 (17.6%)	
In the neighbourhood	-	32 (26.9%)	32 (20.9%)	
Other places	1 (2.9%)	15 (12.6%)	15 (9.8%)	
**Activity engaged in during the bite**				
While farming or harvesting	-	11 (9.2%)	11 (7.1%)	
Collecting firewood	-	5 (4.2%)	5 (3.3%)	
Bathing/cleaning	3 (8.8%)	11 (9.3%)	14 (9.1%)	
Sleeping	19 (55.9%)	1 (0.8%)	20 (13.1%)	
Walking	4 (11.8%)	62 (52.1%)	66 (43.1%)	
Stationary (seated or standing)	7 (20.6%)	5 (4.2%)	12 (7.8%)	
Playing outdoors	-	2 (1.7%)	2 (1.3%)	
While doing manual work	1 (2.9%)	18 (15.1%)	19 (12.4%)	
Fishing	-	2 (1.7%)	2 (1.3%)	
Watering plants	-	2 (1.7%)	2 (1.3%)	
**Month of year**				
January—March	16 (47.1%)	55 (46.2%)	71 (46.4%)	X^2^ = 2.9; df = 3; p = 0.4
April—June	1 (2.9%)	15 (12.6%)	16 (10.5%)	
July—September	9 (26.5%)	25 (21.0%)	34 (22.2%)	
October—December	8 (23.5%)	24 (20.2%)	32 (20.9%)	
**Time of day**				
Day time (6.00 am-6.00 pm)	15 (44.1%)	80 (67.2%)	95 (62.1%)	X^2^ = 6; df = 1; **p = 0.01**
Night-time (6.00 pm-6.00 am)	19 (55.9%)	39 (32.8%)	58 (37.9%)	
**Perceived adequacy of light**				
Yes	14 (41.2%)	63 (52.9%)	77 (50.3%)	X^2^ = 1.5; df = 1; p = 0.3
No	20 (58.8%)	56 (47.0%)	76 (49.7%)	
**Total**	**34 (100%)**	**119 (100%)**	**153 (100.0%)**	

*Only those who had lower limb bites.

#### Person

When considering personal characteristics, outdoor bites were proportionately higher among males, while indoor bites were higher among females. Krait bites occurred more commonly indoors, while Russell viper bites occurred more commonly outdoors; hump-nosed viper bites were the commonest both indoors and outdoors. Non-venomous bites were more common indoors.

Lower limbs were the commonest site bitten in both indoor and outdoor bites. Among them, almost all were barefooted and had clothing above ankles at the time of the bite, with higher proportions seen among indoor bites.

#### Place of occurrence

Snakebites mostly took place outdoors in vulnerable locations such as in the field (paddy fields, chena land), neighbourhood (riverbeds, gravel roads) and immediate home environment (home gardening). Activities engaged in at the time of bite varied according to the type of bite. Most of the outdoor bites took place while walking (52.1%) followed by while doing manual work (15.1%), farming/harvesting (9.2%) and bathing (9.3%). In contrast, the indoor bites took place predominantly while sleeping (55.9%) or being stationary (20.6%).

#### Time of occurrence

Snakebites occurred most commonly within the first three months of the year (46.4%) and outdoor bites were more common than indoor bites during the April-June period. The majority of outdoor bites took place during daytime (67.2%), while indoor bites occurred during night-time (55.9%) (p<0.05). Although there was no difference between indoor and outdoor bites in relation to perceived adequacy of lighting, most of the indoor bites took place under poor light.

## Discussion

This study aimed to assess the true burden of snakebites within rural agricultural communities in the dry agro-climatic zone in Sri Lanka, by conducting a population-based study in the district of Ampara, which has been estimated to have one of the highest prevalence of snakebites. Thus far, this is the largest study from a single district in Sri Lanka screening 8707 residents from 2500 households that included 816 snakebites.

### Extent of the snakebite problem in rural areas

We found that the one-year point prevalence for snakebites in Ampara was 17.6 per 1000 rural residents (95% CI: 15–20.6) and 6.12 per 100 rural households (95% CI: 5.25–7.13). When the lifetime prevalence of snakebite was assessed, our results showed that one in 10 individuals and one in 3 households were susceptible to snakebites. Since both the average bite per person and household were found to be approximately 1, it ruled out a significant effect of clustering of snakebites that could have occurred due to repeated bites in the same individual or in the same household. Notably, the case fatality ratio was exceedingly low. This may reflect improved accessibility and greater utilisation of allopathic medical services in the region, and wide availability of antivenom in hospitals.

In a recently published national survey with geospatial analysis, the annual incidence of snakebite in the Eastern Province was found to be 368 (95% Cl 227–509) per 100,000 [8]. The comparatively higher one-year point prevalence found in our study is attributable to selection of households from more rural regions within Ampara district where snakebites are likely to be more frequent than from the whole province. Furthermore, the previous study sampled only 67 snakebites from the whole of the Eastern Province that includes three districts (Ampara, Batticaloa and Trincomalee), whereas our study included a higher number of snakebites from a single district. The annual incidence of snakebite in our study is higher than that reported in regional countries such as Bangladesh and Nepal [3,5], and second only to that reported from India [6]. These comparisons were consistent with the estimates of the global incidence of snakebites, which denotes Sri Lanka as one of the highest snakebite reporting countries of the world [1].

### Vulnerability of rural residents to snakebite

Although there are many global studies that have described the epidemiological characteristics of snakebite victims [3–6,8,16], the risk imparted by each characteristic of the victims has not been evaluated against non-snakebite victims. Such identification of the exact population groups most vulnerable to snakebites is important for prioritising allocation of health resources including improvement of living conditions within a given high risk area for snakebite. A recent global mapping of snakebite hotspots used the metrics of living within range of medically important snakes, distance to major urban centres and healthcare access and quality index deciles to identify vulnerable populations at area level [25]. A local study too has identified similar parameters using geo- spatial analysis in Sri Lanka [8]. However, these metrics act as generalised correlates that at best can only produce approximate environmental -level estimates for populations, which are superseded for accuracy by studies such as ours that considered multiple factors in determining individual-level vulnerability to snakebites within identified communities (Table 2). Our study identified adult males living alone and having economic hardships as the most vulnerable to snakebites (adjusted OR>2), highlighting vulnerability in individual and social dimensions, leading to lapses in targeted educational programmes on preventive measures through existing social networks within agricultural communities. Furthermore, though all rural residents are equally exposed to snake habitats, those in farming or unskilled occupations were at higher risk after adjusting for confounders, indicating a neglected occupational hazard. Being of Sinhala ethnicity probably reflected the ethnic bias towards farming in Sri Lanka. Similar characteristics of vulnerability to snakebite has been reported from a small qualitative study conducted in Brazil among victims of snakebites [26].

### Indoor versus outdoor bites

Over 75% of snakebites in our study had occurred outdoor similar to the reports from Malaysia, North India, Nepal and previous studies done in Sri Lanka [3,5,8,13,14,27–36]. Our study however is unique in that it has gone on further to compare and analyse the circumstantial characteristics of outdoor and indoor bites to assess the variation of vulnerability factors in relation to person, place and time of snakebites at an individual level. Intriguingly, we found that outdoor bites were more common among males; during daytime; mostly while walking; on lower limbs; during the April-June period reflecting harvesting periods; and the culprit snakes were mostly hump-nosed and Russell vipers, whereas indoor bites were more common among females; during night-time; predominantly while sleeping or being stationary and barefooted; mostly on lower limbs; and the culprit snakes were mostly hump-nosed vipers and kraits. Non-venomous snakebites were more common indoors. The findings suggest the importance of improving living and housing conditions especially sleeping arrangements and housekeeping of rural residents as much as educating them on snakebite. There was no literature available to compare the circumstances related to indoor and outdoor bites.

## Conclusions & recommendations

This study adds to the sparse amount of population-based snakebite prevalence data, and for the first time, characterises the elements of vulnerability of snakebite victims, sampling a sizeable population from a typically rural agricultural community from a district in Sri Lanka known to have a high density of snakebites. The findings of our study can be recommended to inform healthcare decision making and resource prioritisation in developing snakebite safe living environments within regions of human dwellings plagued by venomous snakes. Furthermore, our results are applicable to other countries in Asia with similar epidemiological and environmental characteristics.

### Study limitations

Since snakebite is a strikingly memorable event for a person, they are likely to remember and recall it better than any other event. However, recall bias is a well-known limiting factor for collecting quality data on past events. We minimised this bias as much as possible by eliciting associations only in relation to the bites within the preceding 12 months. There was no bias caused by the protracted survey effort since the recall of snakebites during the preceding 12 months included all climatic seasons in the Ampara District.
